# Serum TFF1 and TFF3 but not TFF2 are higher in women with breast cancer than in women without breast cancer

**DOI:** 10.1038/s41598-017-05129-y

**Published:** 2017-07-07

**Authors:** Yuko Ishibashi, Hiroshi Ohtsu, Masako Ikemura, Yasuko Kikuchi, Takayoshi Niwa, Kotoe Nishioka, Yoshihiro Uchida, Hirona Miura, Susumu Aikou, Toshiaki Gunji, Nobuyuki Matsuhashi, Yasukazu Ohmoto, Takeshi Sasaki, Yasuyuki Seto, Toshihisa Ogawa, Keiichiro Tada, Sachiyo Nomura

**Affiliations:** 1 Department of Breast and Endocrine Surgery, Graduate School of Medicine, The University of Tokyo, Tokyo, Japan; 2 Center of Clinical Sciences, National Center for Global Health and Medicine, Tokyo, Japan; 3 Department of Pathology, The University of Tokyo Hospital, Tokyo, Japan; 4 Breast Center, International University of Health and Welfare, Mita Hospital, Tokyo, Japan; 5 Department of Gastrointestinal Surgery, Graduate School of Medicine, The University of Tokyo, Tokyo, Japan; 6 NTT Medical Center Tokyo, Tokyo, Japan; 7 Otsuka Pharmaceutical Tokusima Research Institute, Tokyo, Japan; 8 Breast Center, Dokkyo Medical University Koshigaya Hospital, Tokyo, Japan

## Abstract

Breast cancer remains a common malignancy in women, but the take-up for breast cancer screening programs in Japan is still low, possibly due to its perceived inconvenience. TFF1 and TFF3 are expressed in both breast cancer tissue and normal breast. Serum trefoil proteins were reported as cancer screening markers for gastric, prostate, lung, pancreatic cancer and cholangio carcinoma. The purpose of this study was to examine whether serum trefoil proteins could be screening biomarkers for breast cancer. Serum trefoil proteins in 94 breast cancer patients and 84 health check females were measured by ELISA. Serum TFF1 and TFF3 were significantly higher and serum TFF2 was significantly lower in breast cancer patients. Area under the curve of receiver operating characteristic of TFF1, TFF2, and TFF3 was 0.69, 0.83, and. 0.72, respectively. AUC of the combination of TFF1, TFF2, and TFF3 was 0.96. Immunohistochemically, TFF1 expression was positive in 56.5% and TFF3 was positive in 73.9% of breast cancers, while TFF2 was negative in all tumors. Serum TFF1 had positive correlation with expression of TFF1 in breast cancer tissue. Serum concentrations of TFF1 and TFF3 but not TFF2 are higher in women with breast cancer than in women without breast cancer.

## Introduction

Breast cancer is one of the most common malignancies in women. In 2014 in Japan, 13,240 women died of breast cancer, which remains the leading cause of cancer death in women. The incidence of breast cancer is still increasing in Japan as well as in the world^1^. Breast cancer screening with mammography or ultrasound is provided for women beginning at 40 years of age by local governments in Japan, however the response rate for the examination is only 34.2% in 2013^2^. Some reasons for this low rate may be embarrassment and discomfort of the examinations. If breast cancer can be screened by a blood test, instead of mammography or ultrasound, the response rate of the screening examinations would increase.

Trefoil factors are small (6–12 kDa) and stable peptides secreted by the mammalian epithelial mucus secreting cells in gastrointestinal tract^3–9^. They were named due to the existence of a common 3 loop leaf-like structure, which makes the peptides extremely stable towards proteolytic digestion as well as acid and heat degradation^8^. Trefoil proteins constitute a family of three peptides (TFF1, TFF2, and TFF3) that are widely expressed in a tissue specific manner in the body^5^. Trefoil proteins are expressed in certain cells in gastrointestinal mucosa^5^. Originally, TFF1 was cloned from MCF-7, a breast cancer cell line^10^. TFF3 expression is predominantly in intestine. However, TFF3 mRNA expression was found in normal mammary gland and breast cancer tissue^11^. The promotor regions of TFF1 and TFF3 have estrogen responsive element^12, 13^. In normal breast tissue, TFF1 and TFF3 are reported to be expressed in human breast epithelium in lower amounts, but there is no expression of TFF2^14^. In breast cancer tissue, TFF1 protein was positive in 68% and TFF3 protein was positive in more than 90% of invasive ductal carcinoma^15^. The positive rate of TFF1 and TFF3 in invasive lobular carcinoma, ductal carcinoma *in situ* (DCIS), and lobular carcinoma *in situ* (LCIS) was reported to be higher than in invasive ductal carcinoma^15–17^.

We have previously reported that serum TFF3 can be used as a screening marker for gastric cancer^18^. It can also be a biomarker for pancreatic cancer (manuscript under preparation). Other studies have reported TFF3 as a biomarker for lung cancer, prostate cancer, and cholangiocarcinoma^19–22^. TFF proteins are also reported as metastatic biomarkers for colon cancer^23^. TFF1 and TFF3 are expressed in breast cancer cells^11^. The expression of TFF1 and TFF3 differs between malignant cells isolated from patients with lung adenocarcinoma and those isolated from patients with breast adenocarcinoma^24^. Detection of TFF1 and TFF3 mRNAs in peripheral blood has been proposed as a surrogate measure of circulating tumor cells^25^. These findings prompted us to analyze whether serum TFF1, TFF2, and TFF3 can be biomarkers for breast cancer^18, 20, 26, 27^. In this study, we have analyzed whether serum TFF1, TFF2, and TFF3 can be biomarkers of breast cancer.

## Results

### Test Set Patients characteristics

Serum was taken from 94 breast cancer patients before treatment. Among them 8 patients were excluded: 3 patients received neo-adjuvant chemotherapy and 5 patients had bilateral breast cancer. The clinicopathological characteristics of the remaining 86 patients were shown in Table 1. The ages of breast cancer patients ranged from 32 to 88 years (average age: 56) and healthy individuals ranged from 26 to 82 years (average: 52).

**Table 1:** Clinicopathological status of breast cancer on serum TFF levels.

Characteristic	Total	Serum TFF1 (ng/ml)	TFF1 P value	serum TFF2 (ng/ml)	TFF2 P value	serum TFF3 (ng/ml)	TFF3 P value
Age (years, mean ± SD)	56 ± 12.5						
Sex							
Women	86						
Menopausal status (missing 1)			0.0008		0.0007		0.284
Premenopausal	35	0.7 ± 0.4		3.1 ± 1.0		6.4 ± 1.1	
Postmenopausal	50	1.4 ± 1.1		4.5 ± 2.1		6.9 ± 2.9	
Histological type			0.823		0.666		0.785
DCIS	12	1.2 ± 1.0		4.3 ± 1.6		6.9 ± 1.5	
IDC	60	1.1 ± 0.9		3.8 ± 1.9		6.8 ± 2.6	
Others	14	1.2 ± 0.9		4.0 ± 1.7		6.3 ± 1.7	
Tumor size (missing 2)		0.224		0.341		0.278	
≤2 cm	54	1.0 ± 0.7		3.8 ± 1.4		6.5 ± 1.7	
>2 cm	30	1.3 ± 1.2		4.2 ± 2.4		7.1 ± 3.2	
Nodal status (missing 7)			0.525		0.555		0.604
pN−	60	1.1 ± 0.8		4.0 ± 1.6		6.7 ± 1.8	
pN+	19	1.3 ± 1.3		3.7 ± 2.6		7.0 ± 3.7	
Nuclear grade (missing 10)			0.021		0.053		0.934
I	42	0.9 ± 0.5		3.6 ± 1.3		6.8 ± 1.8	
II	14	1.7 ± 1.6		5.0 ± 3.1		6.6 ± 3.5	
III	20	1.1 ± 0.8		3.8 ± 1.5		6.6 ± 2.7	
Lymphatic vessel invasion (missing 3)			0.582		0.062		0.733
Negative	62	1.1 ± 1.0		4.1 ± 1.9		6.7 ± 2.2	
Positive	21	1.0 ± 0.7		3.2 ± 1.5		6.5 ± 2.6	
Vascular invasion (missing 3)			0.956		0.315		0.292
Negative	64	1.1 ± 1.0		4.0 ± 1.9		6.8 ± 2.2	
Positive	19	1.1 ± 0.8		3.5 ± 1.3		6.2 ± 2.6	
ER (missing 4)			0.163		0.306		0.096
Negative	10	0.7 ± 0.4		3.4 ± 0.8		5.6 ± 1.8	
Positive	72	1.2 ± 1.0		4.0 ± 1.9		6.9 ± 2.4	
PR (missing 4)			0.679		0.899		0.690
Negative	14	1.2 ± 1.2		4.0 ± 1.5		6.5 ± 2.3	
Positive	68	1.1 ± 0.9		3.9 ± 1.9		6.8 ± 2.4	
HER2 (missing 9)			0.860		0.968		0.430
Negative	71	1.1 ± 0.9		3.9 ± 1.9		6.8 ± 2.4	
Positive	6	1.0 ± 0.8		3.9 ± 1.3		6.0 ± 2.5	
Ki67 (missing 11)			0.363		0.999		0.577
<30%	52	1.1 ± 0.6		3.9 ± 1.6		6.7 ± 1.8	
≥30%	23	1.3 ± 1.4		3.9 ± 2.3		7.0 ± 3.4	
Subtype (missing 11)			0.196		0.643		0.173
LuminalA	47	1.1 ± 0.7		3.9 ± 1.7		6.8 ± 1.7	
LuminalB	20	1.4 ± 1.4		4.1 ± 2.4		7.3 ± 3.6	
HER2 type	3	0.7 ± 0.3		3.1 ± 0.8		4.4 ± 0.7	
Triple negative	5	0.6 ± 0.3		3.1 ± 0.7		5.8 ± 2.1	

The levels of TFFs in serum were compared with the clinicopathological status of breast cancer. Only serum TFF1 was statistically different among nuclear grade, that is nuclear grade II> nuclear grade I, III. SD: Standard Deviation, IDC: Invasive Ductal Carcinoma.

### Serum TFF1, TFF2, and TFF3 levels

**Figure 1:**
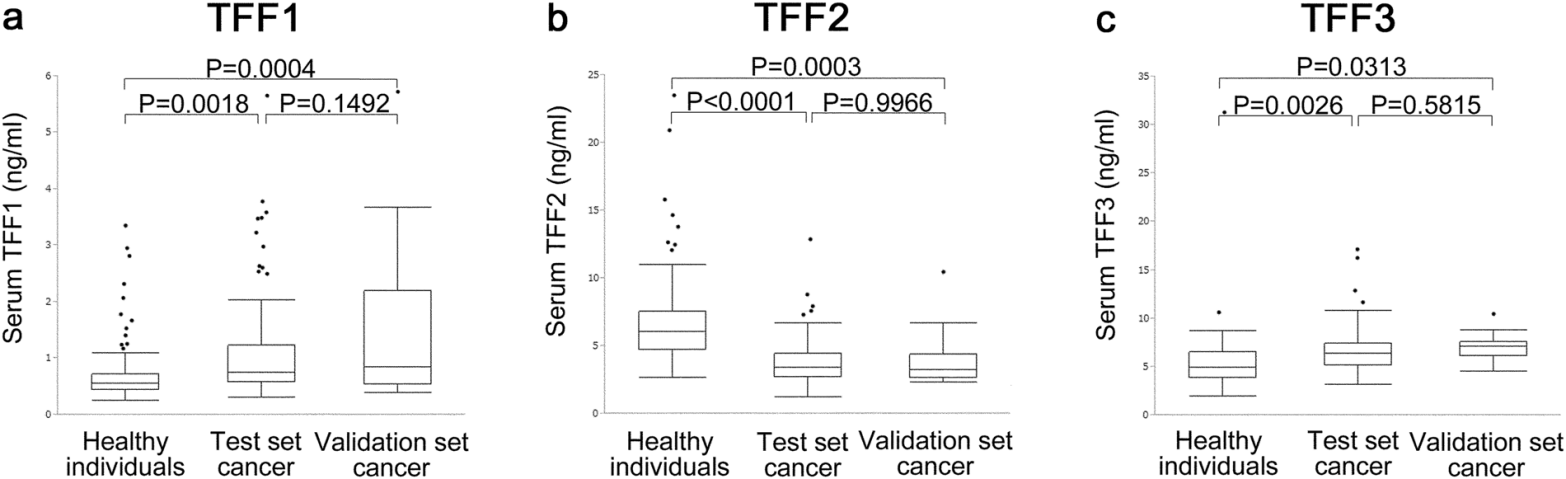
Serum TFF1, TFF2, and TFF3 values of healthy individuals and breast cancer patients. Serum TFF1, TFF2, and TFF3 values (**a**–**c**) of healthy individuals, breast cancer patients in test set, and breast cancer patients in validation set. Serum TFF1 and TFF3 levels in test set breast cancer patient were significantly higher than in healthy individuals (**a** and **c**) (P = 0.0018 and P = 0.0026). Serum TFF2 in test set breast cancer patient level was significantly lower than in healthy individuals (**b**) (P < 0.0001). Similarly, serum TFF1 and TFF3 levels in validation set breast cancer patient were significantly higher than in healthy individuals (**a**,**c**) (P = 0.0004 and P = 0.0313). Serum TFF2 level in validation set breast cancer patient was significantly lower than in healthy individuals (**b**) (P = 0.0003). There was no significant difference in serum TFF1, TFF2 and TFF3 between test set and validation set. Serum TFF (ng/ml) value in the vertical axis.

Serum TFF1, TFF2, and TFF3 in 94 breast cancer patients and 84 healthy individuals were measured and compared. TFF1, TFF2, and TFF3 values were all statistically different between breast cancer patients and healthy individuals (Fig. 1a–c). Serum TFF1 and TFF3 levels in breast cancer patients were significantly higher than in the healthy individuals. Serum TFF2 levels in breast cancer patients were significantly lower than in the healthy individuals (TFF1: 1.12 ± 0.93 ng/ml (breast cancer patients), 0.75 ± 0.59 ng/ml (health individuals), P = 0.0018, TFF2: 3.90 ± 1.80 ng/ml (breast cancer patients), 6.87 ± 3.61 ng/ml (healthy individuals) P < 0.0001, TFF3: 6.68 ± 2.31 ng/ml (breast cancer patients), 5.38 ± 3.32 ng/ml (healthy individuals), P = 0.0026) (Fig. 1a–c).

**Figure 2:**
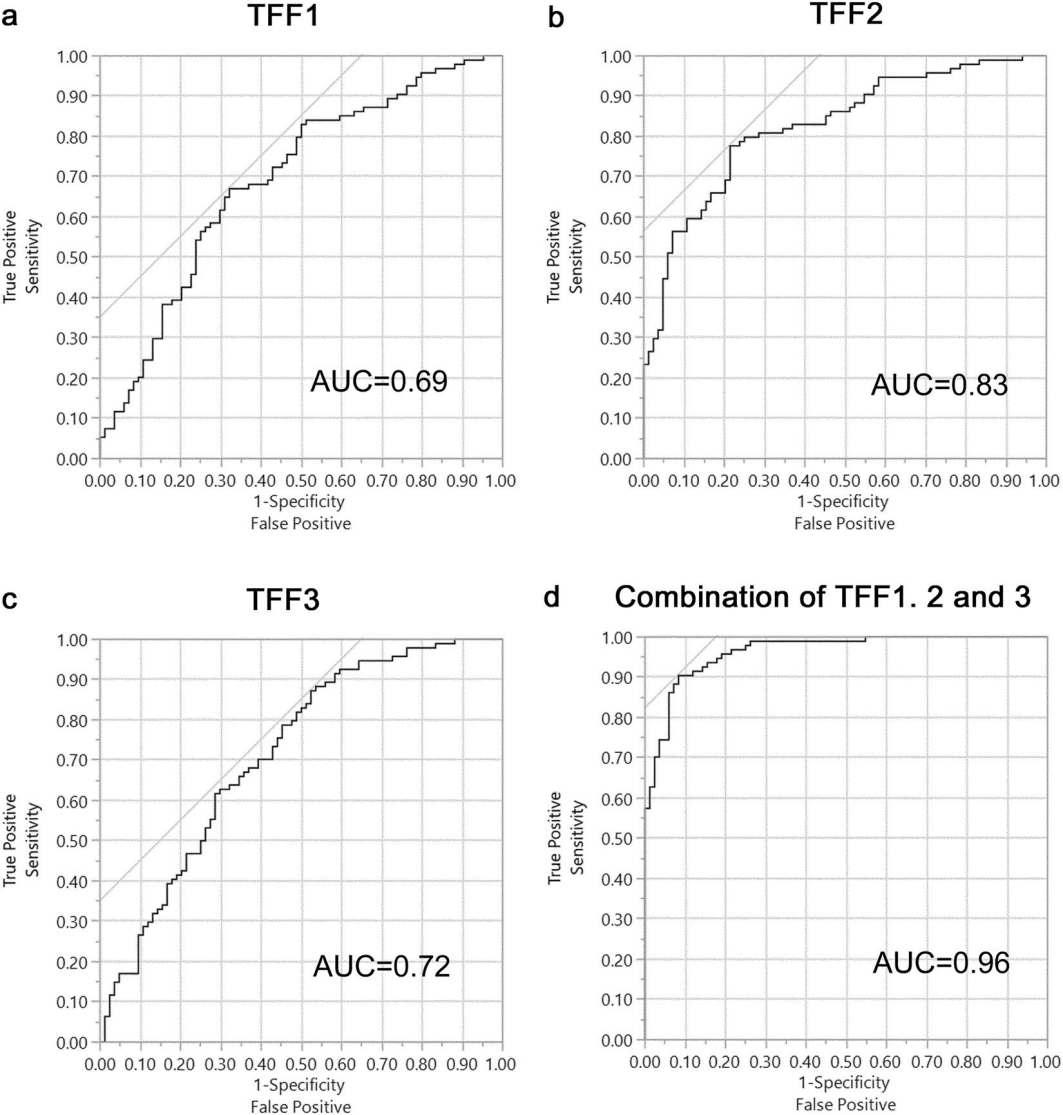
ROC curves of serum TFF1, TFF2, and TFF3 and the combination of TFF1, TFF2, and TFF3. ROC curves of serum TFF1 (**a**), serum TFF2 (**b**), serum TFF3 (**c**), and the combination of TFF1, TFF2, and TFF3 (**d**). AUC for TFF1, TFF2 and TFF3 was 0.69, 0.83 and 0.72. The AUC of the combination of TFF1, TFF2, and TFF3 was 0.96.

To determine the diagnostic accuracy of serum TFF1, TFF2, and TFF3, ROC analysis was performed. AUC for TFF1, TFF2, and TFF3 was 0.69, 0.83, and. 0.72 (Fig. 2a–c), and the order of accuracy for breast cancer patient screening was TFF2, TFF3 and TFF1. Only TFF2 was lower in breast cancer patients compared to healthy individuals. Therefore, the ROC curve for serum TFF2 is the diagnostic accuracy of lower serum TFF2 in breast cancer patients. The AUC of the combination of TFF1, TFF2, and TFF3 was 0.96 and the combination of these tests had a much better AUC (Fig. 2d). Based on these ROC, the cut-off value of TFF1, TFF2, and TFF3 is 0.65 ng/ml, 4.43 ng/ml. and 4.62 ng/ml, respectively. With these cut off, the sensitivity and the specificity of TFF1 are 0.67 and 0.68, these of TFF2 are 0.78 and 0.79, and these of TFF3 are 0.87 and 0.48.

### The serum TFF1, TFF2, and TFF3 levels and histological types of breast cancer

To test the influence of breast cancer characteristics on serum TFF1, TFF2, and TFF3 levels, the levels of TFF1, TFF2, and TFF3 in serum were compared with the clinicopathological status of breast cancer. Table 1 Only serum TFF1 was statistically different among nuclear grade, that is nuclear grade II> nuclear grade I, III. There was no significant difference in serum TFF1, TFF2, and TFF3 levels in any other clinicopathological status of breast cancer including histological types, tumor size, nodal status, nuclear grade, lymphatic invasion, venous invasion, status of ER, status of PR, HER2, Ki67 and molecular subtypes.

### Immunohistochemistry

**Figure 3:**
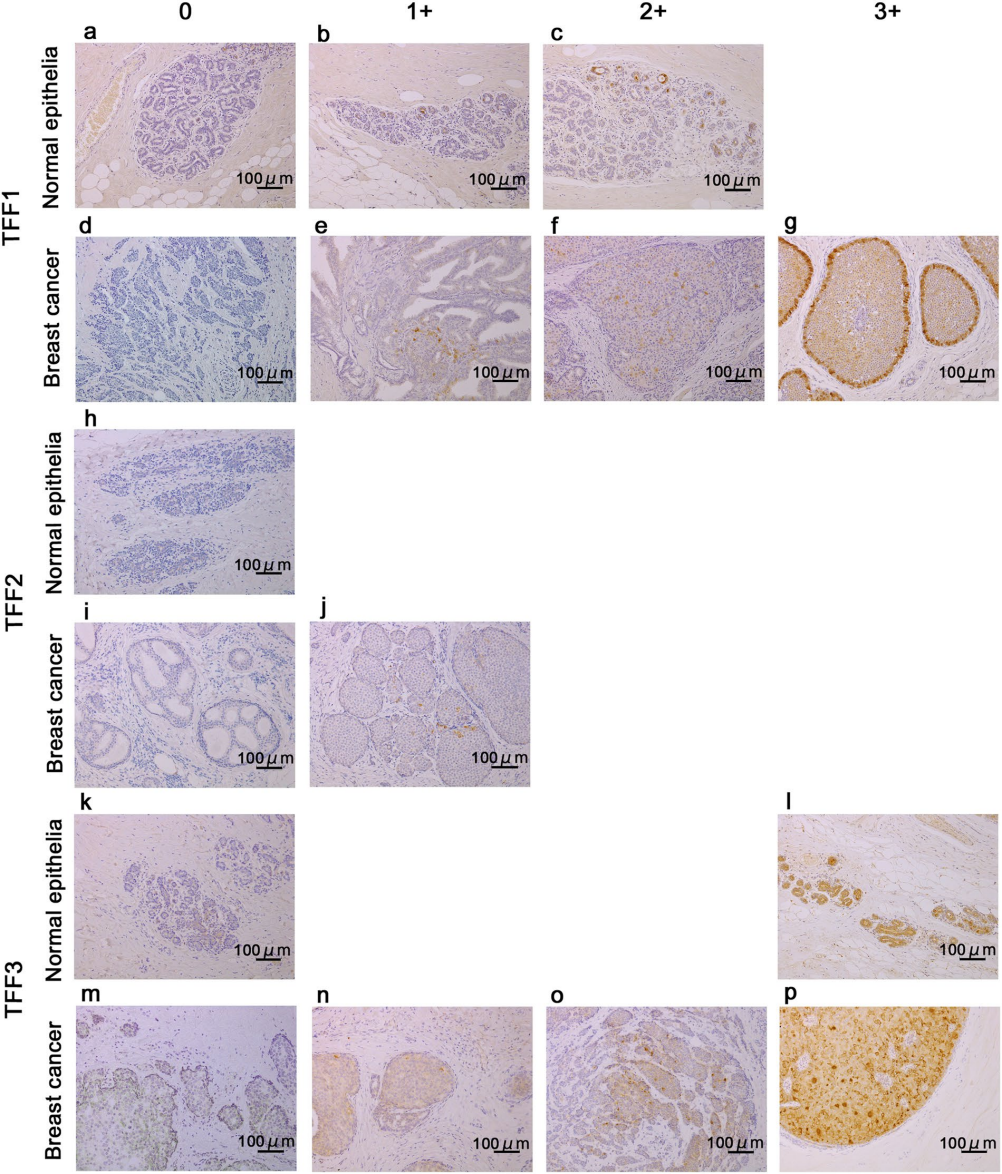
IHC for TFF1, TFF2, and TFF3 for breast cancer tissue and normal epithelial tissue. IHC for TFF1, TFF2, and TFF3 for breast cancer tissue and normal epithelial tissue surrounding breast cancer. Under the view of 10x objective lens, no staining was defined to be -, staining of less than 10% cells to be 1+, 1050% cells to be 2+, more than 50% cells to be 3+. The number of the patients of TFF1 staining in normal epithelial tissue - (**a**), 1+ (**b**) and 2+ (**c**) were 27 (58.7%), 15 (32.6%) and 4 (8.7%), respectively. There was no patient of TFF1 3+ in surrounding normal breast tissue. TFF1 staining in breast cancer tissue - (**d**), 1+ (**e**), 2+ (**f**) and 3+ (**g**) were 9 (19.6%), 11 (23.9%), 20 (43.5%) and 6 (13.0%), respectively. Only 6 (13.0%) cancer was stained by TFF1 weaker than surrounding non-cancerous tissue. TFF2 staining in normal epithelial tissue - (**h**) was 46 (100%), and TFF2 staining in breast cancer tissue - (**i**), 1+ (**j**) were 29 (63.0%) and 17 (37.0%), respectively. TFF3 staining in normal epithelial tissue - (**k**) and 3+ (**l**) were 36 (78.3%) and 10 (21.7%), respectively. There was no patient of TFF3 1+ and TFF3 2+ staining in surrounding normal breast tissue. TFF3 staining in breast cancer tissue - (**m**), 1+ (**n**), 2+ (**o**) and 3+ (**p**) were 2 (4.4%), 10 (21.7%), 10 (21.7%) and 24 (52.2%), respectively. Only 4 (8.7%) cancer was stained by TFF3 weaker than surrounding non-cancerous tissue (Scale bar: 100 μm).

Immunohistochemical staining (IHC) for TFF1, TFF2, and TFF3 was performed for breast cancer tissue and normal epithelial tissue surrounding breast cancer. All trefoil factors were localized cytosolic. Among the 46 breast cancers analyzed, TFF1 staining was graded as 3+ in 6 patients (13%), 2+ in 20 patients (43.5%), 1+ in 11 patients (23.9%), and no staining in 9 patients (19.6%). Overall, 26 out of 46 (56.5%) breast cancer was positive for TFF1. In normal epithelial tissue surrounding breast cancer, 4 (8.7%) patients were positive for TFF1. TFF3 staining was graded as 3+ in 24 patients (52.2%), 2+ in 10 patients (21.7%), 1+ in 10 patients (21.7%), and no staining in 2 patients (4.4%). Overall, 34 out of 46 (73.9%) breast cancer was positive for TFF3. In normal epithelial tissue surrounding breast cancer, 10 (21.7%) patients were positive for TFF3. Importantly, only 6 (13.0%) cancers were stained by TFF1 weaker than surrounding non-cancerous epithelial tissue, and only 4 (8.7%) cancers was stained for TFF3 weaker than surrounding non-cancerous epithelial tissue. On the other hand, immunohistochemical staining of TFF2 graded as 1+ and 0 were 17 (37.0%) and 29 (63.0%) and no tumors were of graded 2+ or 3+. TFF2 was negative in both breast cancer tissue and normal epithelial tissue surrounding breast cancer. The representative staining results are shown in Fig. 3.

### TFF1, TFF2, and TFF3 expression in breast cancer and clinicopathological characteristics

The relation between TFF1, TFF2, and TFF3 expressing cell rate in breast cancer tissue and clinicopathological characteristics is shown in Table 2. TFF1 expression was significantly correlated with PR expression only, however, TFF3 expression was not related to any clinicopahological characteristics.

**Table 2:** Clinicopathological status of breast cancer and immunohistochemical staining (IHC).

Characteristic	Total	TFF1	TFF1 P value	TFF3	TFF3 P value
Age (years, mean ± SD)	54 ± 12.3				
Sex					
Women	46				
Histological type			0.456		0.614
DCIS	5	1.8 ± 0.8		2.4 ± 0.9	
IDC	35	1.3 ± 1.0		2.0 ± 1.0	
Others	6	1.7 ± 1.0		2.3 ± 1.0	
Tumor size			0.534		0.793
>2 cm	17	1.5 ± 0.9		2.1 ± 1.0	
≤2 cm	29	1.3 ± 1.0		2.1 ± 1.0	
Nodal status (missing 3)			0.102		0.904
pN−	29	1.2 ± 0.8		2.1 ± 1.0	
pN+	14	1.7 ± 1.1		2.1 ± 1.0	
Nuclear grade (missing 6)			0.917		0.610
I	25	1.4 ± 0.9		2.0 ± 1.0	
II	5	1.2 ± 1.1		1.8 ± 1.1	
III	10	1.4 ± 1.3		2.3 ± 1.1	
Lymphatic vessel invasion			0.305		0.205
Negative	34	1.5 ± 0.9		2.0 ± 1.0	
Positive	12	1.2 ± 1.1		2.4 ± 0.9	
Vascular invasion			0.719		0.564
Negative	34	1.4 ± 0.9		2.1 ± 1.0	
Positive	12	1.5 ± 1.1		2.3 ± 0.9	
ER (missing 1)			0.109		0.836
Negative	6	0.8 ± 0.8		2.2 ± 1.0	
Positive	39	1.5 ± 1.0		2.1 ± 1.0	
PR (missing 2)			0.011		0.793
Negative	7	0.6 ± 0.5		2.0 ± 1.3	
Positive	37	1.6 ± 1.0		2.1 ± 0.9	
HER2 (missing 6)			0.182		0.342
Negative	36	1.4 ± 1.0		2.0 ± 1.0	
Positive	4	0.8 ± 0.5		2.5 ± 1.0	
Ki67 (missing 7)			0.059		0.650
<30%	28	1.5 ± 0.9		2.1 ± 1.0	
≥30%	11	1.3 ± 1.0		1.9 ± 1.0	
Subtype (missing 7)			0.067		0.253
LuminalA	25	1.7 ± 0.9		2.0 ± 1.0	
LuminalB	10	1.0 ± 0.9		2.0 ± 1.1	
HER2 type	2	0.5 ± 0.7		3.0 ± 0	
Triple negative	2	0.5 ± 0.7		1.0 ± 0	

TFF1 expression was significantly correlated with PR expression only, however, TFF3 expression was not related to any clinicopahological characteristics. SD: Standard Deviation, IDC: Invasive Ductal Carcinoma.

### Association of serum TFF1, TFF2, and TFF3 with immunohistochemical staining

**Figure 4:**
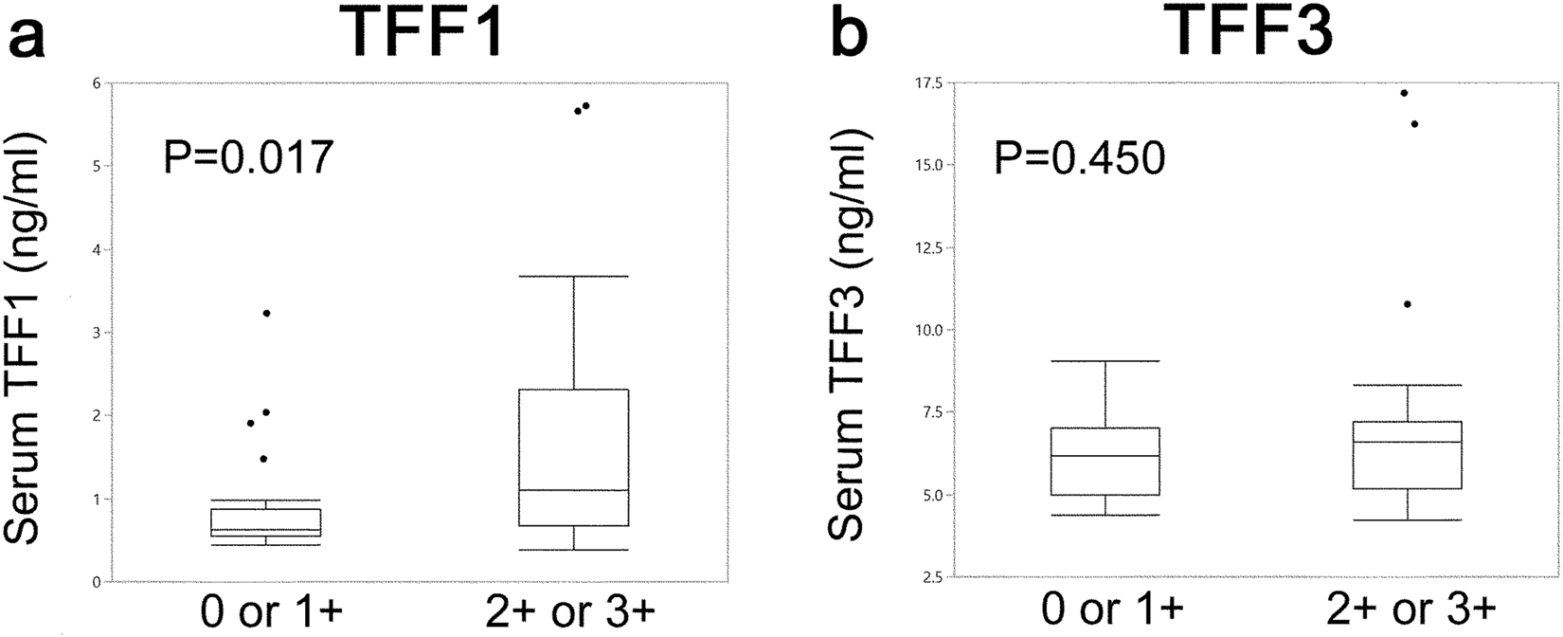
Relations between serum TFF levels and histologically expressing TFF for TFF1 and TFF3. Relations between serum TFF levels and histologically expressing TFF in breast cancer tissue for TFF1 and TFF3 were analyzed (**a**,**b**). The horizontal line is the classification of the immunohistochemically negative (− and 1+) and positive (2+ and 3+) for TFF expression. The vertical line is serum TFF value (ng/ml). The serum TFF1 in patients with histologically TFF1 positive breast cancer was significantly higher than in histologically TFF1 negative breast cancer patients (P = 0.017) (**a**). There was no significant correlation between serum TFF3 and histological positivity of TFF3 expression in breast cancer (P = 0.450) (**b**).

We next examined the correlations between serum TFF1 and TFF3 levels and expression of TFF1 and TFF3 in breast cancer. Serum TFF1 of histologically TFF1 positive breast cancer patients was 1.74 ± 0.26 ng/ml, and that of histologically TFF1 negative breast cancer patients was 0.88 ± 0.23 ng/ml. The serum TFF1 in patients with immunohistologically positive for TFF1 in breast cancer was significantly higher than patients with immunohistologically negative for TFF1. (P = 0.017) (Fig. 4a). However, we could not detect a significant difference of serum TFF3 value between patients with histologically TFF3 positive breast cancer and patients with histologically TFF3 negative breast cancer. (6.92 ± 0.44 ng/ml and 6.26 ± 0.74 ng/ml, P = 0.446) (Fig. 4b).

### Histoscore and serum Trefoil protein

**Figure 5:**
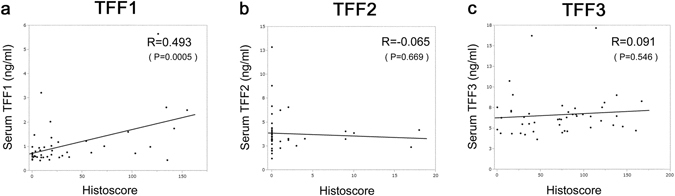
Relations between serum TFF levels and Histoscore. Relations between serum TFF levels and Histoscore of TFF in breast cancer tissue were analyzed. R = 0.493 (p = 0.0005) for TFF1 (**a**), R = 0065 (p = 0.669) for TFF2 (**b**), and R = 0.091 (p = 0.546) for TFF3 (**c**). Only TFF1 showed significant positive correlation.

To confirm the results of the relation of immunohistochemistry and serum TFF, we counted the Histoscore of each section and plotted with serum TFF^28^. Figure 5 Only TFF1 showed significant positive correlation between Histoscore and serum TFF1. TFF2 and TFF3 did not show significant correlations, and the results above were confirmed.

### Validation Set Serum TFF1, TFF2, and TFF3

For evaluation of the serum TFF1, TFF2, and TFF3 for cancer screening, serum of 22 breast cancer patients before treatment were evaluated. All the patients were female and their age was from 41 to 82 years (average age: 60). Serum TFF1, TFF2, and TFF3 all had the same difference as the test sets. Serum TFF l and TFF3 levels in breast cancer patients were significantly higher than in healthy individuals. Serum TFF2 levels in breast cancer patients were significantly lower than in healthy individuals (TFF1: 1.48 ± 1.43 ng/ml, P = 0.0004, TFF2: 3.90 ± 1.90 ng/ml, P = 0.0003, TFF3: 6.96 ± 1.45 ng/ml, P = 0.0313) (Fig. 1a–c).

## Discussion

We have analyzed serum TFF1, TFF2, and TFF3 as screening biomarkers for breast cancer. Serum TFF1 and TFF3 levels in breast cancer patients were significantly higher than in healthy individuals. The serum TFF2 level in breast cancer patients was significantly lower than in healthy individuals. Therefore, the diagnostic accuracy of serum TFF2 is for lower serum TFF2 in breast cancer patients. The combination of the three Trefoil Factor measurements in serum provided a high AUC of 0.96. The serum levels of TFF1, TFF2, and TFF3 did not differ in histological types or clinicopathological characteristics of breast cancer patients. These results suggest that serum TFF1, TFF2, and TFF3 measurement may represent an effective primary screening tool for breast cancer.

Several serum biomarkers for detection of breast cancer have been reported. Lee *et al*. have reported that serum *O-*glycosylated proteins showed 0.869 AUC for stage 0 breast cancer, but it decreased in stage I breast cancer^29^. Vitronectin is also reported to be a possible biomarker for stage 0–1 breast cancer with AUC 0.73. However, it is also inversely correlated to breast cancer stage^30^. Finally, the combination of 5 microRNAs is reported to be a biomarker for breast cancer with 89.7% accuracy^31^. In this report, AUC of combination of serum TFF1, TFF2, and TFF3 is 0.96 suggesting that combined testing for TFF1, TFF2, and TFF3 can provide a powerful tool for breast cancer screening.

Immunohistochemical staining for TFF1 was positive in 56.5% of breast cancer tissues and TFF3 was positive in 73.9% of breast cancer tissues. However, TFF2 was negative in all of the breast cancer tissues. TFF2 expression in breast cancer was contravertial, however, our immunohistochemical staing results were negative (0 and 1+) with 1+ staining for 37.0% breast cancer tissue^32, 33^. These results correlate well to serum trefoil protein changes, and the positive immunostaining rates for TFF1 in breast cancer tissue correlated well with serum TFF1 level. Based on this result, elevated serum TFF1 may originate directly from breast cancers. However, the positive cell immunostaining rates for TFF3 did not correlate well with serum TFF3 levels. It may be because we analyzed the trefoil factor positivity in breast cancer tissue with one representative section, and the entire tumor volume was not available for analysis. Trefoil protein secretion by cells may contribute to the serum concentration of trefoil proteins. Trefoil protein is expressed in gastrointestinal mucosa, and it may be also a sourse of serum trefoil protein. mRNA of TFF1 and TFF3 expression in normal breast tissue was reported previously^15^. In our cohort, TFF1 was positive in normal epithelial tissue surrounding breast cancer in 8.7% of patients and TFF3 was positive in normal epithelial tissue surrounding breast cancer in 21.7% of patients. This may reflect hormonal status, but there was no correlation with age of the patients. Otherwise, the origin of high serum TFF3 is of different mechanisms. We reported that serum TFF1, TFF2, and TFF3 could be good biomarkers for gastric cancer screening^3^. Among them, serum TFF3 was the best marker, though, serum TFF3 did not drop down after gastrectomy, differing from serum TFF1 and TFF2. Origin of high serum TFF3 was speculated other than stomach. The origin of high serum TFF3 in breast cancer patients also has possibility of different organ from breast cancer.

The reason for lower serum TFF2 level in breast cancer patients is not known. Trefoil proteins were reported to have positive expressing correlation in gastrointestinal mucosa^34^. However, this is the first report of inverted correlation of a serum Trefoil factors to cancer. There could be control systems of total Trefoil factors in body and higher serum TFF1 and serum TFF3 may affect a reduction of serum TFF2. Further analysis is needed. Trefoil factors are reported to have an estrogen responsive element and TFF1 and TFF3 expressions are correlated to ER positivity in breast cancer^16^. Furthermore, tissue expression of TFF1 and TFF3 could be biomarkers of hormone therapy^13, 35^. However, there was no co-relation between TFF1, TFF2, and TFF3 expression and ER in breast cancer tissue in this patients’ series. This may be because of small size of the samples.

The effects of TFF1 and TFF3 for cancer progression is still controversial. TFF1 knockout mouse showed gastric tumor and TFF1 was believed to be tumor suppressor gene^36^. However, there is a report that TFF1 expression was correlated to worse prognosis^37^. TFF3 expression is also reported to correlate to worse prognosis in aromatase inhibitor treated ER+ breast cancer^38^. TFF1 and TFF3 have an estrogen responsive element, and bad prognosis of TFF1 and/or TFF3 positive breast cancer may be masked by good prognosis of ER+ breast cancer^15^. *In vitro* experiments with breast cancer cell lines, indicate that TFF1 and TFF3 expression promotes migration and invasion whereas their effects on proliferation are controversial^39^.

We previously reported that serum TFF1, TFF2, and TFF3 can be screening biomarkers for gastric cancer^3^. TFF3 is reported to be a biomarker for lung cancer, prostate cancer, and cholangiocarcinoma^4–7^. For practical use of serum TFF1, TFF2, and TFF3 for cancer screening, subjects who were detected to have high serum TFF3, should be scrutinized by imaging examination. At that time, high serum TFF1 and low TFF2 can be used for more refining of breast cancer patients for the imaging examination. After refining, breast cancer is examined by ultrasound or mammogram.

## Conclusions

In summary, this study has demonstrated that the serum TFF1, TFF2, and TFF3 could be effective markers for breast cancer screening. The combination of all 3 trefoil factors provides the best biomarker sensitivity for breast cancer. Serum screening with TFF1, TFF2, and TFF3 for breast cancer can increase the screening receiving rate.

### Limitation

The limitations of this study is that small sample size of the cohorts. And, validation set was compared to the same healthy individuals for the test set. Further analysis of large cohorts will be needed for defining cut off value. And denying the tumor volume in analyzing correlation between TFF1, TFF2, and TFF3 expressions in breast cancer tissue and serum TFF1, TFF2, and TFF3 is also a limitation.

## Methods

### Patients

For the test set, serum from 94 breast cancer patients before treatment was taken. Among them, 59 patients were treated in the University of Tokyo Hospital, 22 patients were treated in the International University of Health and Welfare Mita Hospital, and 13 patients were treated in the Breast Center, Dokkyo Medical University Koshigaya Hospital, from May 2014 to June 2015. All the patients were female and their ages ranged from 32 to 88 years (average age: 56). Their cancer characteristics, histological types, tumor size, nodal status, nuclear grade, lymphatic invasion, venous invasion, status of estrogen receptor (ER), status of progesterone receptor (PR), Human epidermal growth factor receptor 2 (HER2), proliferative activity (Ki67) and molecular subtypes were analyzed. For the validation set, serum from 22 breast cancer patients before treatment, who visited the University of Tokyo Hospital from July 2015 to December 2015 was taken. All the patients were female and their age was from 41 to 82 years (average age: 60).

As healthy individuals, serum was taken from 84 female subjects, who received health checks in the NTT East Japan Kanto Hospital from July 2009 to January 2011. They had no history of malignancy previously or at the time of the health check. Their ages ranged from 26 to 82 years (average: 52). Both breast cancer patients and health check subjects were confirmed to have a normal creatinine level, since TFF1, TFF2, and TFF3 are secreted in urine.

Surgical specimens from 59 breast cancer patients, who were treated in the University of Tokyo Hospital, were collected. Thirteen specimens were not suitable for histological analysis and 46 specimens were used for further analysis. These 46 patients did not have bilateral breast cancer and did not receive pre-operative chemotherapy.

This study was approved by the Institutional Review Board of the University of Tokyo (#10427). Written informed consent was obtained from all participants in accordance with the Declaration of Helsinki and its later revision. All methods were performed in accordance with the guidelines and regulations approved by the Research Ethics Committee of the University of Tokyo.

### Immunoassays

We measured serum TFF1, TFF2, and TFF3 by enzyme-linked immunosorbent assay (ELISA). These ELISA systems were constructed in our laboratory, and the precise information was reported in our previous publication^18^. Briefly, polyclonal antibodies TFF1, TFF2, and TFF3 coated onto 96-well microtiter plates were blocked with 0.1% Bovine serum albumin (BSA) in phosphate buffered saline (PBS). Then the blocking solution was removed, and 100 μl of assay buffer (1 M NaCl, 0.1%BSA, PBS) was added to each well. And then 50 μl of the samples or standard human TFF1, TFF2, and TFF3 were added to the wells. After incubation overnight at room temperature, the plates were washed, and diluted Biotin labeled anti-TFF polyclonal antibodies were added to each appropriate well. After incubation for 2 hours, the plate was washed, and diluted Horseradish peroxidase (HRP) conjugated Streptavidin (Vector) was added to each well. After incubation for 2 hours at room temperature, the plates were washed, and 3,3′,5,5′-tetramethylbenzidine (TMB) solution (Scytek Laboratories, INC.) was added. After incubation for 10 minutes at room temperature, Stop solution (H_2_SO_4_) was added, and the absorbance at 450 nm was measured. Concentrations of human TFF1, TFF2, and TFF3 in the samples were calculated from the standard curves of recombinant human TFF1, TFF2, and TFF3.

### Immunohistochemistry

Immunohistochemistry staining (IHC) analysis of TFF1, TFF2, and TFF3 for breast cancer tissue and normal tissue surrounding breast cancer was performed. Deparaffinized sections were blocked with 1.5% goat serum provided in the Vectastain ABC KIT (Vector Laboratories; Burlingame, CA). TFF2 and TFF3 staining required antigen retrieval with incubation in citric acid buffer solution of pH 6.0 for 20 minutes at 98 °C. Sections were incubated with rabbit polyclonal antibody raised in our laboratory (TFF1: OPP-22201 and TFF3: OPP-22305)^18^. TFF2: mouse IgM αTFF2 kindly provided by Dr. Nick Wright^40^. We confirmed the sensitivity of this antibody^41–44^. The dilutaion was 1:1000, 1:100 and 1:1000 for anti-TFF1, anti-TFF2 and anti-TFF3, respectively) overnight at 4 °C followed by incubation with a biotinylated secondary antibody and peroxidase-conjugated streptavidin (Vectastain ABC KIT, Vector Laboratories, Burlingame, CA). Chromogen was developed with diaminobanzidine. Sections were counterstained with Mayer’s hematoxylin and mounted. Sections were viewed and photographed on an Olympus BX-51 bright-field microscope equipped with a Nikon Digital Sight DS-L imaging system. The sections were analyzed by two pathologists (M. I. and T. S.). Under the view of a 10x objective lens, staining of more than 50% cancer cells was defined to be 3+, 10–50% to be 2+, less than 10% to be 1+, and no staining to be 0. We assigned 0 and 1+ as negative, and 2+ and 3+ as positive for staining.

### Histoscore analysis

To confirm the results of the relation between staining of sections and serum Trefoil protein, we perform modified Histoscore analysis^28^. Histoscore (H-score) was calculated by a semiquantitative assessment of both the intensity of staining (graded as: 0, non-staining; 1, weak; 2, median; or 3, strong using adjacent normal mucosa as the median) and the percentage of positive cells. The range of possible scores was from 0 to 300. Then we plotted the Histoscore and serum TFF values.

### Statistical analysis

The receiving operating characteristics (ROC) and the area under curve (AUC) was used to measure the discriminatory ability of the model. We plotted Receiver Operating Characteristics Curve (ROC curve) 1-specificity and sensitivity of serum Trefoil protein concentration in cancer patiets, and calculated area under curve (AUC). The means of variables were compared between two groups by a t-test. A two-sided P value of less than 0.05 was considered statistically significant. All statistical analyses were performed using JMP11 software (SAS Institute, Cary, NC, USA).
